# The effects of beta-blockers on dobutamine-atropine stress echocardiography: early protocol *versus *standard protocol

**DOI:** 10.1186/1476-7120-4-30

**Published:** 2006-07-19

**Authors:** Ana C Camarozano, Plínio Resende, Aristarco G Siqueira-Filho, Luis H Weitzel, Rosangela Noe

**Affiliations:** 1 Cardiology Department, Barra D'or Hospital, Rio de Janeiro, Brazil, National Heart Institute, Rio de Janeiro, Brazil; 2 Cardiology Department, Barra D'or Hospital, Federal University of Rio de Janeiro, Rio de Janeiro, Brazil; 3 Internal Medicine Department, University Hospital, Federal University of Rio de Janeiro (UFRJ), Rio de Janeiro, Brazil; 4 Cardiology Department, National Heart Institute, Rio de Janeiro, Brazil; 5 Statistical Department, Federal University of Rio de Janeiro, Rio de Janeiro, Brazil

## Abstract

**Background:**

To study the effects of Beta-blockers during Dobutamine Stress Echocardiography (DSE) comparing the hemodynamic benefits of an early administration of atropine in patients taking or not Beta-blockers.

**Methods:**

One hundred and twenty-one patients were submitted to dobutamine stress echocardiography for the investigation of myocardial ischemia. The administration of atropine was randomized into two groups: A or B (early protocol when atropine was administered at 10 and 20 mcg/kg/min of dobutamine, respectively) and C (standard protocol with atropine at 40 mcg/kg/min of dobutamine). Analysis of the effects of Beta-blockers was done regarding the behavior pattern of heart rate and blood pressure, test time, number of conclusive and inconclusive (negative sub-maximum test) results, total doses of atropine and dobutamine, and general complications.

**Results:**

Beta-blocked patients who received early atropine (Group A&B) had a significantly lower double product (p = 0.008), a higher mean test time (p = 0.010) and required a higher dose of atropine (p = 0.0005) when compared to the patients in this group who were not Beta-blocked. The same findings occurred in the standard protocol (Group C), however the early administration of atropine reduced test time both in the presence and absence of this therapy (p = 0.0001). The patients with Beta-blockers in Group A&B had a lower rate of inconclusive tests (26%) compared to those in Group C (40%). Complications were similar in both groups.

**Conclusion:**

The chronotropic response during dobutamine stress echocardiography was significantly reduced with the use of Beta-blockers. The early administration of atropine optimized the hemodynamic response, reduced test time in patients with or without Beta-blockers and reduced the number of inconclusive tests in the early protocol.

## Background

The beta receptors are distributed in the heart leading to an increase of the inotropism and chronotropism. They are found in the smooth muscle (vascular and bronchial) causing dilatation and relaxation of these structures, and a considerable number is also found in the cardiac muscle exerting positive inotropic and chronotropic influence [1]. By definition the Beta-blockers are substances which antagonize in a specific, competitive and reversible manner the action of the endogenous or exogenous catecholamines in the beta adrenergic receptors. Thus, the blocker occupies the same receptor site as the agonist, reducing its response [2].

Since these agents are often administered to patients referred to stress echocardiography and are not interrupted before the test, information about the effects of beta-adrenergic blocking on the physiological response of a dobutamine echocardiogram is important.

Previous studies [3] associated atropine to the dobutamine echocardiogram in an attempt to provide a more effective strategy to increase heart rate (HR) with the resulting increase of oxygen consumption by the myocardium. The authors concluded that after the addition of atropine there was an increase in the sensitivity of the test.

From then on, the dobutamine-atropine protocol became known as a safe method with excellent diagnostic accuracy [4-6]. The co-administration of the atropine sulphate became popular in the Beta-blockers era, as the anti-angina drugs pronouncedly reduce the sensitivity of the stress echocardiogram, both with dipyridamole [6] and dobutamine [7]. The same does not seem to occur in the dobutamine-atropine stress echocardiography. A plausible explanation is the fact that the atropine may compensate the chronotropic deficit caused by the Beta-blockers [7].

Beta-blockers may improve or even prevent stress induced wall abnormalities [8,9], implying in a decrease of the ischemic response. As a result, the failure in obtaining an adequate HR which allows for a diagnostic test (conclusive) reduces the precision of the method and seems not to correspond to the plasmatic dose of dobutamine, whose seric level shows a dose-effect relation [10].

The value of the dobutamine-atropine early protocol among patients, Beta-blocked or not, is still speculative. Therefore our objective was to study the influence of Beta-blockers in the early and standard protocols, and compare the potential hemodynamic benefits of the early administration of atropine among patients taking Beta-blockers or not.

## Methods

One hundred and twenty-one consecutive patients examined and referred to DSE were studied during a one year period to evaluate coronary artery disease (CAD).

The patients were randomly divided into Groups A, B and C, where Groups A and B were considered belonging to the early protocol (with atropine administration at 10 and 20 mcg/kg/min of dobutamine, respectively) and group C belonging to the standard protocol (atropine at 40 mcg/kg/min of dobutamine).

We have previously shown the feasibility and accuracy of early protocol of administration of atropine during dobutamine stress echocardiography [11]. When compared to the standard one, the early protocol is faster and diagnostically more accurate [11].

Patients with counter-indication for the test were excluded from this study according to exclusion criteria such as: patients with acute myocardium infarction ≤ 10 days, history of angina < 48 hours and investigation of myocardial viability.

Demographic characteristics, risk factors, use of medication, and ECG, ECHO & angiographic data from total sample are shown on Table 1.

**Table 1:** Demographic characteristics, risk factors, medication, and ECG, Echo and Angiographic data according to Groups.

**VARIABLES**	**GROUP A (n = 41)**	**GROUP B (n = 35)**	**GROUP C (n = 45)**	***p value***
Age	60,8 +/- 11	59,6 +/- 12	54 +/- 12	**0,038**
Gender (male %)	51,2	54,2	65	0,47
Reasons for test:				
-Investigation for CAD (%)	46	66	55	0,23
-Evaluation of known CAD (%)	54	34	45	0,23
Hypertension (%)	61	62	45	0,61
Diabetes (%)	20	20	21	0,98
Hyperlipemia (%)	46	62	35	0,10
Family history for CAD (%)	49	60	46	0,49
Smoking (%)	29	31	24	0,80
Obesity (%)	36	28	45	0,40
Use of Beta-blocker (%)	49	54	45	0,74
Use of Calcium blocker (%)	12	20	10	0,48
Altered baseline ECG (%)	38	39	41	0,97
Altered baseline Echo (%)	41	37	42	0,87
Normal LV function (%)	66	86	65	0,097
Previous angiography (%)	46	40	36	0,68
Previous PTCA (%)	19	12	10	0,60
Previous CABG (%)	17	17	10	0,69

CAD = coronary artery disease, ECG = electrocardiogram,

Echo = echocardiogram, LV = left ventricle, PTCA = percutaneous transluminal coronary angioplasty,

CABG = coronary artery bypass graft.

The present study was approved by the Ethical and Research Committee of the hospital and patients' informed consent was obtained.

The left ventricle was divided into 16 segments [12]. Each segment was described as: normal, hypokinetic, akinetic or dyskinetic according to the American Society of Echocardiography.

We considered inconclusive tests those where HR was lower than 85% of the maximum expected at the end of the protocol (negative sub-maximum test).

During the exam a 3-lead ECG was maintained, as well as continuous monitoring of HR and blood pressure at the end of each stage, before and after the administration of atropine.

Two kinds of equipment were employed in this study: a HDI 5000 from ATL (digitalization – "Image View"), and a Sonos 5500 from HP, both with 4,0 MHz transducers.

### Statistical analysis

The *X^*2 *^test *or the *Fisher exact test*s were used for the comparison of proportions between continuous variables. The *Student t *test was applied for independent samples to compare the means between the two groups as well as the *Mann-Whitney test *(non-parametric test) whenever the variable did not present normal distribution.

The *Analysis of Variance *(ANOVA) was used to compare the means between the groups when all three groups had to be analyzed. The *Analysis of Variance of Kruskal-Wallis *(non-parametric test) was used for the comparative analysis of variables which did not present a normal distribution.

The adopted criteria for determining any significance was set at 5%, in other words, whenever the value of *p *of the statistical analysis was ≤ 0.05.

## Results

Of the total sample (n = 121), 76 patients received early atropine (Groups A & B). Not all 45 patients in the standard protocol group received atropine, since this depended on the end-point of the test.

Of the total number of patients studied, 45% were taking Beta-blockers and there was no difference among the groups regarding the use of this drug.

**Figure 1:**
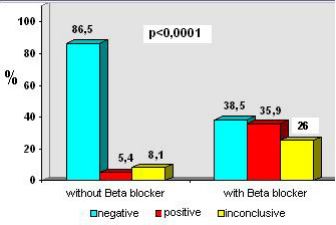
Test result according to Beta-blocker for Group A & B (early protocol).

**Figure 2:**
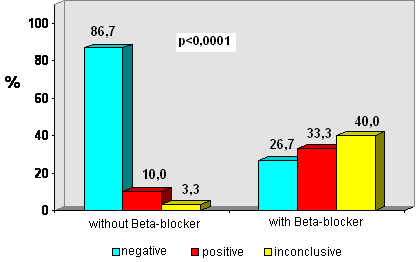
Test result according to Beta-blocker for group C (standard protocol).

In patients taking Beta-blockers, inconclusive results predominated in the standard protocol group (40%) when compared to the early protocol (26%) as shown in the Figures 1 and 2.

**Figure 3:**
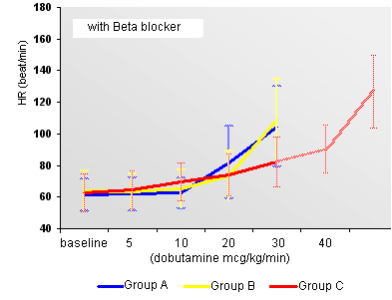
Behavior of HR during use of Beta-blocker in the early and standard protocols.

**Figure 4:**
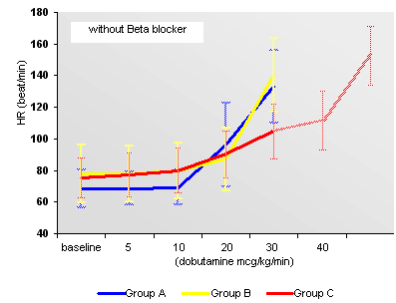
Behavior of HR in the absence of Beta-blocker in the early and standard protocols.

**Figure 5:**
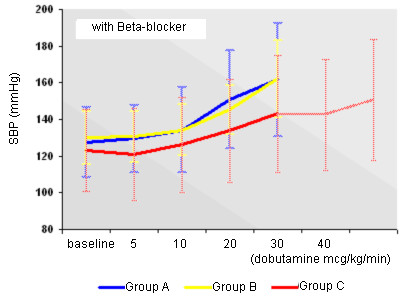
Behavior of SBP during use of Beta-blocker in the early and standard protocols.

**Figure 6:**
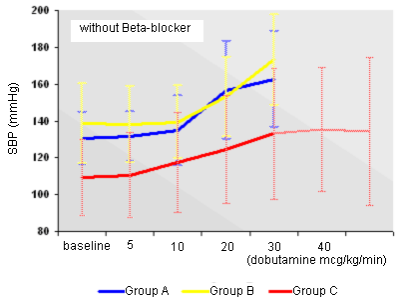
Behavior of SBP in the absence of Beta-blocker in the early and standard protocols.

Behavior of HR with or without Beta-blockers may be seen in Figures 3 and 4. Behavior of SBP with or without Beta-blockers may be seen in Figures 5 and 6.

Descriptive analysis of ATR dose, test time, HR × SBP and DOB dose, according to Beta-blocker, for groups A & B (early protocol) or group C (standard protocol) can be seen in Tables 2 and 3, respectively.

**Table 2:** Descriptive analysis of ATR dose, test time, HR × SBP and DOB dose, according to Beta-blocker, for Groups A + B (early protocol).

**Variables**	**Beta**	**n**	**Mean**	***p *Value**
ATR Dose	No	37	0,64 ± 0,37	0,0005
	Yes	39	1,04 ± 0,54	
Test time	No	37	11,95 ± 2,88	0,010
	Yes	39	13,77 ± 3,19	
HR × SBP	No	37	24674 ± 4308	0,008
	Yes	39	21520 ± 5702	
DOB Dose	No	34	19 ± 4	0,055
	Yes	37	23 ± 4	

**Table 3:** Descriptive analysis of ATR dose, test time, HR × SBP and DOB dose, according to Beta-blocker, for standard protocol (Group C).

**Variables**	**Beta**	**n**	**Mean**	***p *Value**
ATR Dose	No	30	0,38 ± 0,49	0,005
	Yes	15	1,03 ± 0,80	
Test time	No	30	15,83 ± 3,55	0,024
	Yes	15	18,73 ± 4,59	
HR × SBP	No	30	21566 ± 5445	0,028
	Yes	15	17991 ± 3877	
DOB Dose	No	25	19 ± 4	0,089
	Yes	11	25 ± 6	

Table 4 shows the level of significance of the statistical analysis for results, complications and interruption of test. We observed that patients not taking Beta-blockers presented a significantly higher proportion of negative results than those taking Beta-blockers. On the other hand, patients taking Beta-blockers showed significantly higher positive or inconclusive results compared to the patients without Beta-blockers. There was no difference between early and standard protocols in relation to complications.

**Table 4:** Level of significance of the statistical analysis (*p *value) for results, complications and interruption of test.

**Variables**	**Group A**	**Group B**	**Group C**
Results	0,001	0,010	0,005
General complications	0,14	0,64	0,37
Interruption of test	0,15	0,17	0,18

General complications: supra-ventricular arrhythmia, ventricular arrhythmia, hypotension, chest pain, hypertension.

Table 5 shows the test accuracy for the early (A+B) and standard (C) protocol in patients who were submitted to angiography.

**Table 5:** Test accuracy for Groups A+B *versus *C (n = 22).

Frequency Results	A+B	C	Total
neg	3	1	4
	20.00	14.29	
Pos	12	6	18
	80.00	85.71	
Total	15	7	22

neg = negative test pos = positive test

## Discussion

Many patients did not reach the desired HR during DSE. Possible explanations described in literature may justify this fact such as, chronotropic incompetence [13], paradox sinus deceleration [14], and the Bezold-Jarisch reflex [15,16]. However, the most common reason was the use of Beta-adrenergic blockers.

In a study conducted by Weissman et al.[9], the authors found evidence that the hemodynamic parameters such as: HR, arterial pressure, area of left ventricle and coronary blood flow increased more slowly and to a lower degree in the presence of Beta-blockers, which resulted in a prolonged test time. The necessary dobutamine dose in the Beta-blocked group was also significantly higher than in the group with no adrenergic blocking [9].

Therefore it is important to observe the accuracy of the test during the use of Beta-blockers. Sawada et al.[17] demonstrated that the sensitivity was similar between patients who were Beta-blocked and those who were not, however the authors suggest a possible bias in the selection of patients (more severe CAD in Beta-blocked patients). Other authors believe the low HR peak in the presence of Beta-blockers adversely affects the sensitivity of the method [9,18], thus recommending the increase of the dobutamine dose or the assistance of other drugs such as atropine [19-21].

In the current analysis, a significant decrease in the total dose of dobutamine was detected after early administration of atropine and there was no need to increase the dose of cholinergic antagonist. Besides, the hemodynamic response during the dobutamine test was pronouncedly reduced in the presence of Beta-blocking therapy, most likely due to its protective effect on the induction of myocardium ischemia.

In addition, the blood pressure response was higher in patients who were on Beta-blocking therapy and did not receive early atropine. Vatner et al.[22] also presented evidence in which Beta-blockade caused substantial raise in the arterial blood pressure and a slight increase in HR in patients who used dobutamine. The authors believe that this fact involves the combination of the vago baroreceptor activation and a direct blockade on chronotropism, as well as the peripheral action of the drug which releases alpha receptors [22]. Even though these drugs are employed in the treatment for hypertension, they initially cause an increase in the resistance of peripheral vessels. The mechanism controlling hypertension is still not completely clear, but seems to result from the decrease in both the cardiac output and the release of plasmatic Rennin [1].

In this review, the Beta-blocked group presented a lower double product, a higher test time and a greater need for atropine compared to those who were not taking similar anti-ischemic therapy. Another significant finding was the fact that more negative results occurred in patients who were not Beta-blocked. The explanation for a greater number of positive tests in Beta-blocked patients lies in the higher probability of these patients suffering from CAD leading to pharmacologic treatment. However, when we analyze the groups receiving early atropine compared to standard atropine administration, we observed that the early administration of atropine optimized the hemodynamic response of the Beta-blocked group, as well as reduced test time and number of inconclusive results. The chronotropic response exhibited a significant and evident increase soon after initiating the administration of atropine, which was maintained on an increasing scale up to the end of test. Even though atropine did not completely abolish the effects of the Beta-blockers, its addition allowed for a more conclusive test, implying in a higher possibility of inducing myocardial ischemia, a finding also in agreement with the literature [19,23].

Furthermore we observed that the number of inconclusive tests in Beta-blocked patients who were submitted to the standard protocol was higher than in the early protocol group. Other studies also confirm that the number of inconclusive tests is due to the impossibility of obtaining an adequate HR in these patients [24].

We have previously shown that adverse effects did not increase in the early protocol [11] and others researches demonstrated a lower incidence of arrhythmia and general complications with the use of early protocol [25,26].

In the current analysis both time and hemodynamic response were significantly improved with the early administration of atropine. In the presence of an inhibited hemodynamic response, patients with moderate or significant coronary stenosis may not develop a new wall abnormality. For this reason, if the level of plasmatic catecholamine is already high at a dose of 20 or 30 μg/kg/min [10] and HR does not rise at the same rate, simply increasing infusion time of the drug would not lead to the desired hemodynamic impact, and may imply a cost of extra time.

In the group of Beta-blocked patients, the addition of atropine should compensate the adrenergic blockade effect by increasing HR, and what is more important, increasing the sensitivity for the detection of myocardial ischemia by the echocardiogram [7], thus equalizing the test response.

## Conclusion

Both the hemodynamic and chronotropic response during DSE were pronouncedly decreased with the use of Beta-blockers. Test time and the need for atropine were higher than in the patients with no Beta-blockade. However, the early administration of atropine improved the hemodynamic response and reduced test time in both groups, with or without Beta-blockers, as well as the number of inconclusive tests in the early protocol.

## Competing interests

The author(s) declare that they have no competing interests.

## Authors' contributions

ACC designed and wrote the manuscript. AGSF, PR and LHW performed the revisions and instructions. RN reviewed the statistical analyses of the paper. All authors read and approved the final manuscript.
